# Dataset of benthic foraminiferal community structure from sediment eDNA of Sundarbans mangrove ecosystem

**DOI:** 10.1016/j.dib.2025.111554

**Published:** 2025-04-17

**Authors:** Arkaprava Mandal, Anwesha Ghosh, Gauraw Kumar, Punyasloke Bhadury

**Affiliations:** Integrative Taxonomy and Microbial Ecology Research Group, Department of Biological Sciences, Indian Institute of Science Education and Research Kolkata, Mohanpur, Nadia 741246, West Bengal, India

**Keywords:** Mangrove, Benthic foraminifera, Sediment eDNA, ONT Sequencing

## Abstract

Sundarbans, the world's largest contiguous mangrove wetland, a UNESCO World Heritage Site and a RAMSAR site formed on the delta of Ganga-Brahmaputra-Meghna (GBM) and influenced by coastal water entering from the Bay of Bengal contribute immensely to biodiversity and blue economy. To track the changes driven by natural and anthropogenic stressors, sediment based environmental DNA (eDNA) biomonitoring of benthic foraminifera communities, an important biological group sensitive to changes, has been initiated along with estimation of dissolved nutrients from estuarine surface water of Sundarbans. In pre-monsoon of 2022 (June), sediment cores and surface water were collected as well as *in situ* environmental parameters were measured from two pre-designated sampling points of Sundarbans to elucidate the benthic foraminifera community based on sediment eDNA approach. Based on Oxford Nanopore Technologies (ONT) sequencing in MinION platform, high abundance of *Sorites* sp., *Elphidium excavatum, Textularia gramen, Quinqueloculina* sp. and *Trochammina hadai* were detected. The increasing nutrient concentrations and elucidated benthic foraminiferal signals can contribute towards tracking the state of ecological health of Sundarbans. This study is aimed at generating baseline information on mangrove benthic foraminifera communities using sediment eDNA based high-throughput sequencing.

Specifications Table

**Table:** 

Subject	Earth & Environmental Sciences
Specific subject area	Benthic Ecology
Type of data	Raw and analyzed
Data collection	During sample collection*, in-situ* environmental parameters were measured in triplicate using handheld probes with automatic temperature compensation (ATC). Surface water samples were collected in 1 L wide-mouth amber bottles and preserved with buffered 4% formalin for subsequent analysis of dissolved nutrients. Sediment samples were collected using a handheld corer (corer dimensions: length-10 cm and diameter- 3.5 cm). All the collected sediment samples were immediately fixed with molecular-grade absolute ethanol to undertake eDNA extraction, in addition to grain size analysis.
Data source location	Sundarbans Biological Observatory Time Series (SBOTS) Site, Sagar Island Region: Sundarbans Country: India Latitude and Longitude: Stn1_SB_1 (21°40′45.40"N, 88°8′50.70"E) Stn3_SB_3 (21°40′47.10"N, 88°9′14.60"E)
Data accessibility	Repository name: Bioproject number PRJNA1214056 submitted to NCBI Data identification number: Direct URL to data: https://www.ncbi.nlm.nih.gov/sra/PRJNA1214056

## 1 Value of the Data

• The datasets on benthic foraminifera community from sediment eDNA is the first record from a mangrove environment from Asia and can serve as a basis for detecting changes in foraminifera community structure induced by natural and anthropogenic stressors.
• The generated dataset can act as a biological proxy for assessing changes on biodiversity and linked blue economy for Sundarbans
• The generated sequence data adds to the available global sequence database of foraminifera thereby providing functional understanding of these organisms from data deficient geographical regions.
• The study provides genetic and microscopy-based information on benthic foraminifera communities that can be useful for studying their biogeographic patterns across mangroves
• The dataset will be crucial for scientific community and ecosystem managers towards developing targeted restoration of mangrove ecosystems.

## 2 Background

Foraminifera, a group of testate organisms found in marine environment, are widely used as bioindicators to assess the impacts of changing climate across a wide array of environmental settings [1,2]. Recently, there has been a growing global interest in mapping benthic foraminiferal community structure and developing the efficacy of this group as robust ecological proxy for environmental monitoring using high-throughput sequencing (HTS) [3,4]. However, profiling of foraminifera in mangrove ecosystems using HTS remains poorly mapped, particularly from the context of shallow tropical and subtropical coastal ecosystems spread across Global South. Over the last few decades, Sundarbans mangrove has experienced significant environmental changes including rise in sea levels, increase in frequency of cyclones, storm surges, increased erosion and increase in flow of pollutants. Detailed profiling of benthic foraminiferal communities using sediment eDNA metabarcoding could be crucial to enhance our understanding the consequences of natural and anthropogenic stressors on the health of mangroves such as for Sundarbans. This study focuses on sediment eDNA metabarcoding to characterize benthic foraminiferal community structure from Sundarbans and the generated information along with imaging of foraminifera can contribute towards a deeper understanding of ecological health of mangroves.

### 2.1 Data description

**Fig. 1:**
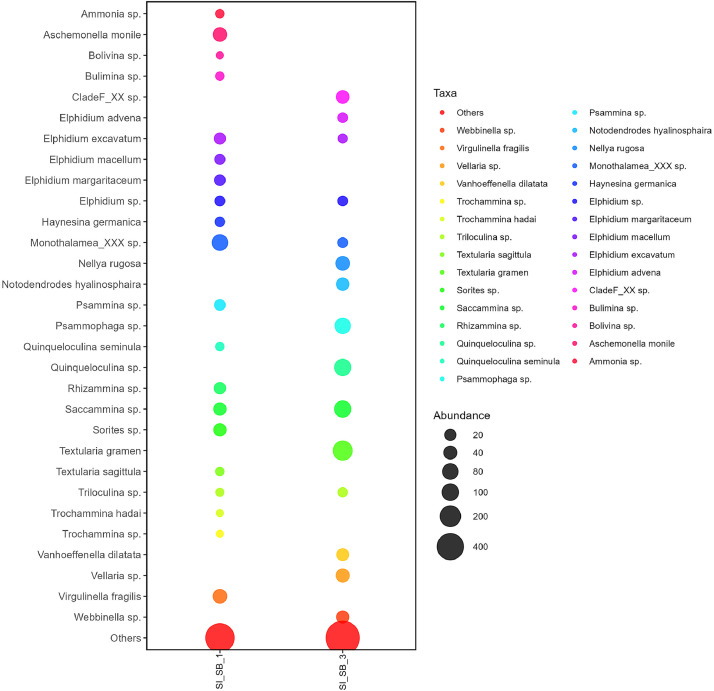
Location of the sampling points representing SBOTS in Sagar Island, which lies along the confluence of the Mooriganga River and the Bay of Bengal.

The dataset presented in this study explains the benthic foraminiferal community structure derived from sediment eDNA collected at two sampling points within the Sundarbans Biological Observatory Time Series (SBOTS) in the Sundarbans (Fig. 1). The dataset also includes measured *in-situ* environmental parameters, dissolved nutrient concentrations, and sediment grain size. The measured environmental parameters are detailed in Table 1**.** The sampling points (Stn1_SB_1 and Stn3_SB_3) are part of pre-designated decadal old time series program site (Sundarbans Biological Observatory Time Series) [[5], [6]] located in southeast of Sagar Island, the largest island of Sundarbans. Sagar Island is influenced by freshwater influx and coastal water on a semi-diurnal basis. Due to enormous freshwater flow and shallow depth, there is high load of suspended particulate matter (SPM) in estuaries surrounding Sagar Island.

**Table 1:** *In situ* environmental parameters measured from surface water and sediment collected from SBOTS site of Sagar Island.

Environmental Parameters	Stn1_SB_1	Stn3_SB_3
Air temperature (⁰ C)	30.8 ±0	29.9±0
Surface water temperature (⁰ C)	30.8±0.1	29.9±0.1
pH	8±0.1	7.9±0
Salinity	17.2±0.06	17.5±0.06
Dissolved oxygen (mg L^−1^)	4.5±0.05	4.9±0.06
Dissolved nitrate (µM)	34.62±0.16	36.94±0
Dissolved ammonia (µM)	1.70±0.0	0.73±0.0
Dissolved *o*-phosphate (µM)	1.04±0.05	1.30±0.05
Dissolved reactive silicate (µM)	74.54±0	62.27±0
Coarse Sand (%)	24.44	17.18
Sand (%)	2.58	3.26
Silt- Clay (%)	72.98	79.56

**Fig. 2:**
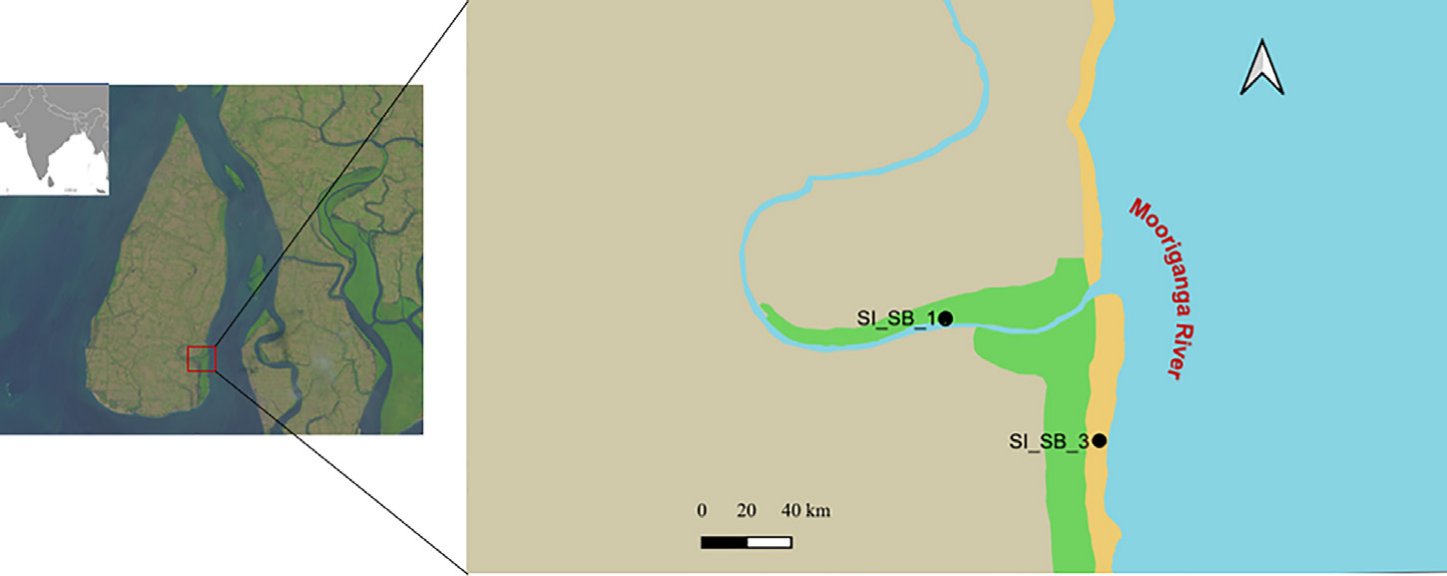
Representation of abundant benthic foraminifera taxa found in Stn1_SB_1 and Stn3_SB_3 based on the analysis of sediment eDNA.

**Fig. 3:**
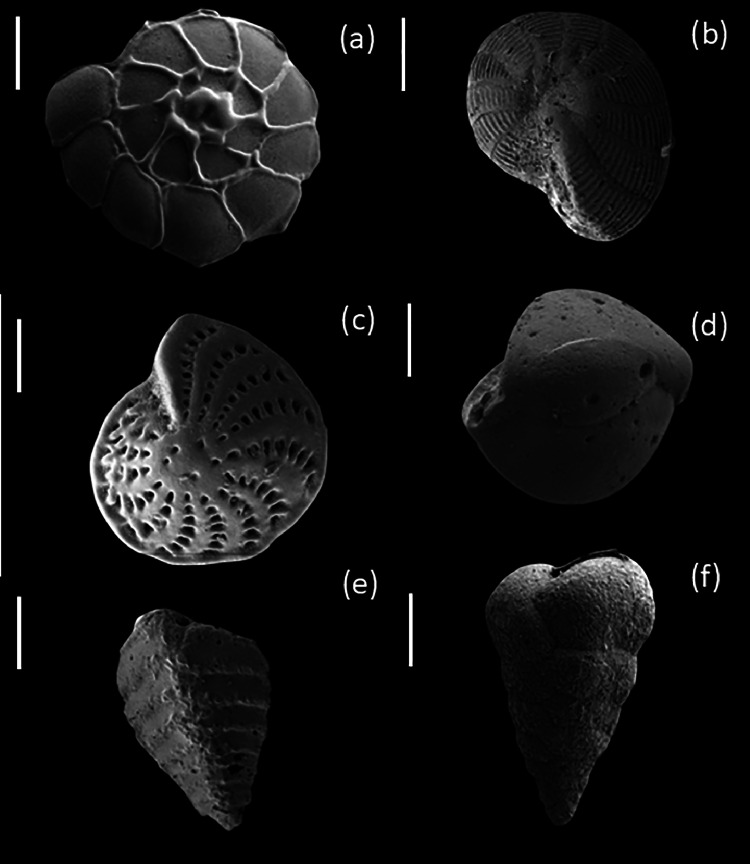
Plate showing FESEM images of representative benthic foraminifera taxa: *Ammonia* sp. a-100 µm; *Elphidium* spp. b and c -100 µm and 20 µm; *Quinqueloculina seminula* d -100 µm; *Textularia* spp. e and f - 100 µm and 20 µm.

Sediment eDNA was extracted in triplicates using HiPurATM Soil DNA purification kit (Himedia, India). The 18S rRNA was amplified in triplicates using uniF and uniR primers. The pooled PCR products were sequenced in a MinION platform Oxford Nanopore Technologies platform (MinION). Approximately, 110 Mb raw data was generated that was cleaned up by removing adapters and barcodes. The quality-controlled data was then annotated against benthic foraminiferal ribosomal reference dataset (BFR2) to identify benthic foraminiferal communities from both sampling points. Stn1_SB_1 showed a high abundance of *Monothalamea*_XXX sp., *Sorites* sp., *Elphidium excavatum, Elphidium advena*, and *Rhizammina* sp. (Fig. 2). Stn1_SB_1 revealed notable presence of calcareous taxa such as *Ammonia* sp., *Bolivina* sp., and *Quinqueloculina seminula*. Besides, agglutinated taxa such as *Textularia sagittula* and *Trochammina hadai* were also encountered*. Textularia gramen, Saccammina* sp., *Quinqueloculina* sp., and *Elphidium advena* were dominant at Stn3_SB_3 (Fig. 2). The presence of these foraminifera identified using sediment eDNA was cross-verified through imaging. Bright-field microscopy, followed by FESEM, showed the presence of these foraminifera genera in studied samples. The FESEM images of representative benthic foraminiferal specimens are shown in Fig. 3. The dataset is available in NCBI under BioSample accession numbers SAMN46357200 and SAMN46357201.

## 3 Experimental Design, Materials and Methods

### 3.1 Study site

The study points are part of Sundarbans Biological Observatory Time Series (SBOTS), a decadal old long-term ecological monitoring program initiated in Sundarbans [details in 5,6]. SBOTS is the only dedicated mangrove ecological time series of Asia. The sampling was conducted in Sagar Island, largest island of the Sundarbans, encompassing an area of approximately 300 km², with an average elevation of 6.7 m above sea level and a mean tidal amplitude ranging from 3.5 to 6.0 m throughout the year [7]. Sagar Island is geographically distinct, bordered by the River Mooriganga to the east, the River Hooghly to the north and west, and the Bay of Bengal to the south [4]. The study was undertaken in close proximity to two predefined sampling stations of SBOTS encompassing the sedimentary layers, Stn1_SB_1 and Stn3_SB_3 (Fig. 2). Stn1_SB_1 is situated within Chemaguri creek and characterized by its close proximity to mangroves, while Stn3_SB_3 is located in the Mooriganga estuary and subject to significant semi-diurnal influence from coastal water of the Bay of Bengal as well as inputs from anthropogenic pollutants originating upstream of the rivers.

### 3.2 Sampling

Sampling was conducted in Sagar Island during pre-monsoon season of 2022 (June) from two sampling points: Stn1_SB_1 (21°40′45.40"N, 88°8′50.70"E) and Stn3_SB_3 (21°40′47.10"N, 88°9′14.60"E). From each point, 1 L of surface water was collected and immediately fixed with buffered 4% formaldehyde (Merck, Germany) to estimate dissolved nitrate (NO_3_^−^), ammonia (NH_4_^+^), *o*-phosphate (PO_4_^3−^), and reactive silicate (SiO_4_^2−^) concentrations following published methodologies [5]. During low tide, sediment samples were collected using a handheld corer (corer dimensions: length-10 cm and diameter- 3.5 cm). Sediment samples were collected for undertaking sediment eDNA extraction and sediment texture analysis. All the collected samples for sediment eDNA extraction were immediately fixed with molecular-grade absolute ethanol (Merck, Germany). The collected water and sediment samples were taken to the laboratory for downstream analyses.

### 3.3 Measurement of *in situ* environmental parameters

During the time of sampling, *in situ* environmental parameters namely, air temperature (AT) and surface water temperature (SWT) (Digital thermometer, Eurolab, Belgium), dissolved oxygen (DO; LUTRON DO-5509, Taiwan), salinity (Oakton Salt 6+Eutech Instrument Pte Ltd., Singapore), and pH (Oakton Eco Testr pH2 Eutech Instrument Pte Ltd., Singapore) were measured in triplicates following standardized methods [6].

### 3.4 Measurement of dissolved nutrient concentrations

Surface water samples were filtered through 0.22 µm (25 mm) nitrocellulose syringe filters (Whatman Uniflo) and subsequently concentrations of dissolved nitrate (NO₃⁻), ammonia (NH₄⁺), *o*-phosphate (PO₄³⁻), and reactive silicate (SiO₄²⁻) were determined. Measurements were performed in triplicates using a UV-Vis spectrophotometer (U-2900, Hitachi, Japan) following published validated protocols [8].

### 3.5 Sediment grain size analysis and imaging of benthic foraminifera

Five grams of unfixed sediment was wet-sieved through stacked sieves with mesh sizes of 500, 250, 63, and 45 µm. The retained sediment fractions in each mesh size were dried and weighed using an analytical balance (Symmetry PA 220 VAC; Cole-Parmer, USA). The weights were subsequently converted to percentages to estimate sediment grain size (Wentworth grain size classes) [9]. Benthic foraminifera were weight sieved through stacked sieves of 500 and 63 µm mesh sizes from 10 cc (wet weight); subsequently >63 µm fraction was retained. Taxon level dentification was performed by wet-picking representative specimens from >63 µm sieved fraction and imaging was performed under a field emission scanning electron microscope (FESEM, Zeiss SUPRA55VP, CarlZeiss AG, Germany).

### 3.6 Sediment eDNA extraction and nanopore sequencing

Sediment samples were collected from two sampling points (Stn1_SB_1 and Stn3_SB_3) of the SBOTS. Approximately, 100 cc of sediment was collected from the 10 cm of depth using a using a handheld corer. Samples were stored at −20°C until further processing. Sediment eDNA was extracted using HiPurATM Soil DNA purification kit (Himedia, India). The extracted sediment eDNA was run on 1% agarose gel. The DNA concentration was quantified using a Nanodrop 2000c Spectrophotometer (Thermo Scientific, USA) and Qubit 1X dsDNA HS (High sensitivity) Assay kit (Thermofisher Scientific, USA). The 18S rRNA region was amplified using UniF (ACCTGGTTGATCCTG) and UniR (TGATCCTTCYGCAGG) primers (GCC BIOTECH, India) from extracted eDNA. PCR conditions included initial denaturation at 95°C for 5 min, 35 cycles of denaturation at 95°C for 30 second, annealing at 58°C for 30 sec, and extension at 72°C for 2 min, followed by a final extension at 72°C for 5 min. Library preparation was performed using Oxford Nanopore Technologies (ONT) ligation sequencing chemistry, involving the SQK-LSK109 ligation sequencing kit (Oxford Nanopore Technologies, Oxford, UK) and EXP-PCR096 native barcoding kit (Oxford Nanopore Technologies, Oxford, UK). The sequencing was performed in the Nanopore MinION (Oxford Nanopore Technologies, Oxford, UK) using SpotON Flowcell R9.4 (FLO-MIN106). Base-calling and demultiplexing of raw reads in FASTQ format were carried out using Guppy v2.3.4 (available at https://community.nanoporetech.com).

### 3.7 Data analyses

The ONT-generated raw reads were concatenated using the ``Concatenate multiple datasets'' tool in the Galaxy platform (usegalaxy.eu)**.** Adapter trimming of the ONT reads was performed using Porechop [10], while quality control was conducted utilizing NanoPlot [11] and Filtlong [12]. QC resulted in 52675 and 35477 high-quality reads that were assembled using MEGAHIT with default parameters [13]**.** Taxonomic classification of assembled sequences was performed using the benthic foraminiferal ribosomal reference dataset (BFR2)**.** Reads were mapped to reference sequences using BLASTn, applying an identity threshold of ≥90–96% for taxonomic assignment and an e-value cutoff of ≤1e-5 (0.00001) [14]. Statistical analyses were conducted in R 3.6.2 using the ggplot2 package for visualization [[15], [16]].

## Limitations

Not applicable.

## Ethics Statement

The work described above did not involve human or animal subjects; therefore, no regulatory compliance guidelines were applicable.

## Data Availability

NCBISediment eukaryotic communities of Sundarbans (Original data).
